# Insulin-like peptide 8 (Ilp8) regulates female fecundity in flies

**DOI:** 10.3389/fcell.2023.1103923

**Published:** 2023-01-18

**Authors:** Haomiao Li, Xi Luo, Na Li, Tao Liu, Junzheng Zhang

**Affiliations:** ^1^ MARA Key Laboratory of Surveillance and Management for Plant Quarantine Pests, College of Plant Protection, China Agricultural University, Beijing, China; ^2^ Chinese Academy of Inspection and Quarantine, Beijing, China

**Keywords:** Ilp8, female fecundity, sexual attractiveness, *Drosophila*, *Bactrocera dorsalis*

## Abstract

**Introduction:** Insulin-like peptides (Ilps) play crucial roles in nearly all life stages of insects. Ilp8 is involved in developmental stability, stress resistance and female fecundity in several insect species, but the underlying mechanisms are not fully understood. Here we report the functional characterization of Ilp8s in three fly species, including *Bactrocera dorsalis*, *Drosophila mercatorum* and *Drosophila melanogaster*.

**Methods:** Phylogenetic analyses were performed to identify and characterize insect Ilp8s. The amino acid sequences of fly Ilp8s were aligned and the three-dimensional structures of fly Ilp8s were constructed and compared. The tissue specific expression pattern of fly Ilp8s were examined by qRT-PCR. In *Bactrocera dorsalis* and *Drosophila mercatorum*, dsRNAs were injected into virgin females to inhibit the expression of Ilp8 and the impacts on female fecundity were examined. In *Drosophila melanogaster*, the female fecundity of Ilp8 loss-of-function mutant was compared with wild type control flies. The mutant fruit fly strain was also used for sexual behavioral analysis and transcriptomic analysis.

**Results:** Orthologs of Ilp8s are found in major groups of insects except for the lepidopterans and coleopterans, and Ilp8s are found to be well separated from other Ilps in three fly species. The key motif and the predicted three-dimensional structure of fly Ilp8s are well conserved. Ilp8 are specifically expressed in the ovary and are essential for female fecundity in three fly species. Behavior analysis demonstrates that Ilp8 mutation impairs female sexual attractiveness in fruit fly, which results in decreased mating success and is likely the cause of fecundity reduction. Further transcriptomic analysis indicates that Ilp8 might influence metabolism, immune activity, oocyte development as well as hormone homeostasis to collectively regulate female fecundity in the fruit fly.

**Discussion:** Our findings support a universal role of insect Ilp8 in female fecundity, and also provide novel clues for understanding the modes of action of Ilp8.

## Introduction

The insulin signaling pathway is highly conserved among metazoans, which plays crucial roles in metabolic homeostasis, growth control, reproduction and lifespan (Chowanski et al., 2021). Orthologs of insulin, known as insulin-like peptides (Ilps) have been identified in insects and the number of the members in the Ilp family are quite variable among different species (Wu and Brown, 2006). In two dipteran species, fruit fly and mosquito, eight Ilps have been characterized in each (Riehle et al., 2006; Nässel et al., 2015). Four genes encoding Ilps are found in the genome of red flour beetle (Li et al., 2008), while only three Ilp genes have been isolated in the locusts (Badisco et al., 2008; Veenstra et al., 2021). Interestingly, approximate thirty-eight Ilps have been found in silkworm (Aslam et al., 2011; Mizoguchi and Okamoto, 2013). Despite the diversified number and type of Ilps, the core components and signal transduction modes of the Ilp signaling pathways are well conserved in the insects (Erion and Sehgal, 2013; Kannan and Fridell, 2013; Nässel and Vanden, 2016; Semaniuk et al., 2021).

Studies using the fruit fly, *Drosophila melanogaster*, have made significant contributions to our understanding of how insect Ilps perform such diversified and critical functions (Chowanski et al., 2021). Among the eight *Drosophila* insulin like peptides (dIlps), dIlp1-6 are believed to act through the same insulin receptor (Nässel and Vanden, 2016), and dIlp7 is recognized by the leucine rich repeat containing G protein receptor Lgr4 (Imambocus et al., 2021). The functions of dIlp1-7 are centered on sensing and responding to nutrition status to meet the metabolic demands in nearly all life stages (Nässel and Vanden, 2016; Chowanski et al., 2021). However, the latest member of dIlps, dIlp8, seems to regulate distinct processes independent of other Ilps (Colombani et al., 2012; Garelli et al., 2012). Recent studies have revealed a unique role of dIlp8 in coordinating larval tissue growth with the onset of metamorphosis (Gontijo and Garelli, 2018). In response to various growth perturbations, fly larval imaginal disc cells produce dIlp8 to communicate the abnormal status with a subset of Lgr3-expressing neurons in the larval brain (Colombani et al., 2015; Garelli et al., 2015; Vallejo et al., 2015). Activation of the dIlp8-Lgr3 pathway eventually leads to a reduction in ecdysone hormone production in the larval endocrine prothoracic gland, thus delaying the onset of metamorphosis (Jaszczak et al., 2016). Current model indicates that dIlp8 signaling extends the larval growth period to allow the regeneration of damaged tissues, and simultaneously slows down the growth rates of healthy tissues to guarantee proportionate development (Gontijo and Garelli, 2018; DaCrema et al., 2021). A role of dIlp8 as interorgan signaling molecule has also been found in the pupae stage during metamorphosis (Ray and Lakhotia, 2019; Heredia et al., 2021).

In contrast to the extensive analysis of the dIlp8-Lgr3 signaling cascade during development, the physiological roles of dIlp8 are less studied. A recent study showed that fly tumor cells secret dIlp8 to suppresses food intake through inhibiting the expression of orexigenic neuropeptides (Yeom et al., 2021). Manipulation of *dIlp8* expression level in adult flies increases resistance to starvation, no matter whether *dIlp8* is mutated or over-expressed (Liao and Nässel, 2020). The *dIlp8* mutant flies display ovulation defect and reduction of female fecundity (Liao and Nässel, 2020). Orthologs of dIlp8 have been described under the name of gonadulin in several insect species, as RNA sequencing analyses suggest that they are highly expressed in the gonads (Veenstra, 2020; Leyria et al., 2022). Knock-down of *Ilp8/gonadulin* expression by RNAi leads to oocyte growth inhibition in the migratory locust (Veenstra et al., 2021) as well as ovulation defect in the kissing bug (Leyria et al., 2022). These observations suggest that Ilp8 might be generally involved in regulation of insect metabolism and reproduction, but the underlying mechanisms are not fully understood.

Here we report the identification and characterization of Ilp8s in three fly species. Our results demonstrate that although the primary amino acid sequences of Ilp8s are variable, the predicted three-dimensional structure and expression pattern are conserved. We provide evidence that Ilp8s are crucial for female fecundity in all three fly species and show for the first time that Ilp8 regulates female sexual attractiveness during the mating process in the fruit fly.

## Results

### Identification and characterization of fly Ilp8s

**FIGURE 1:**
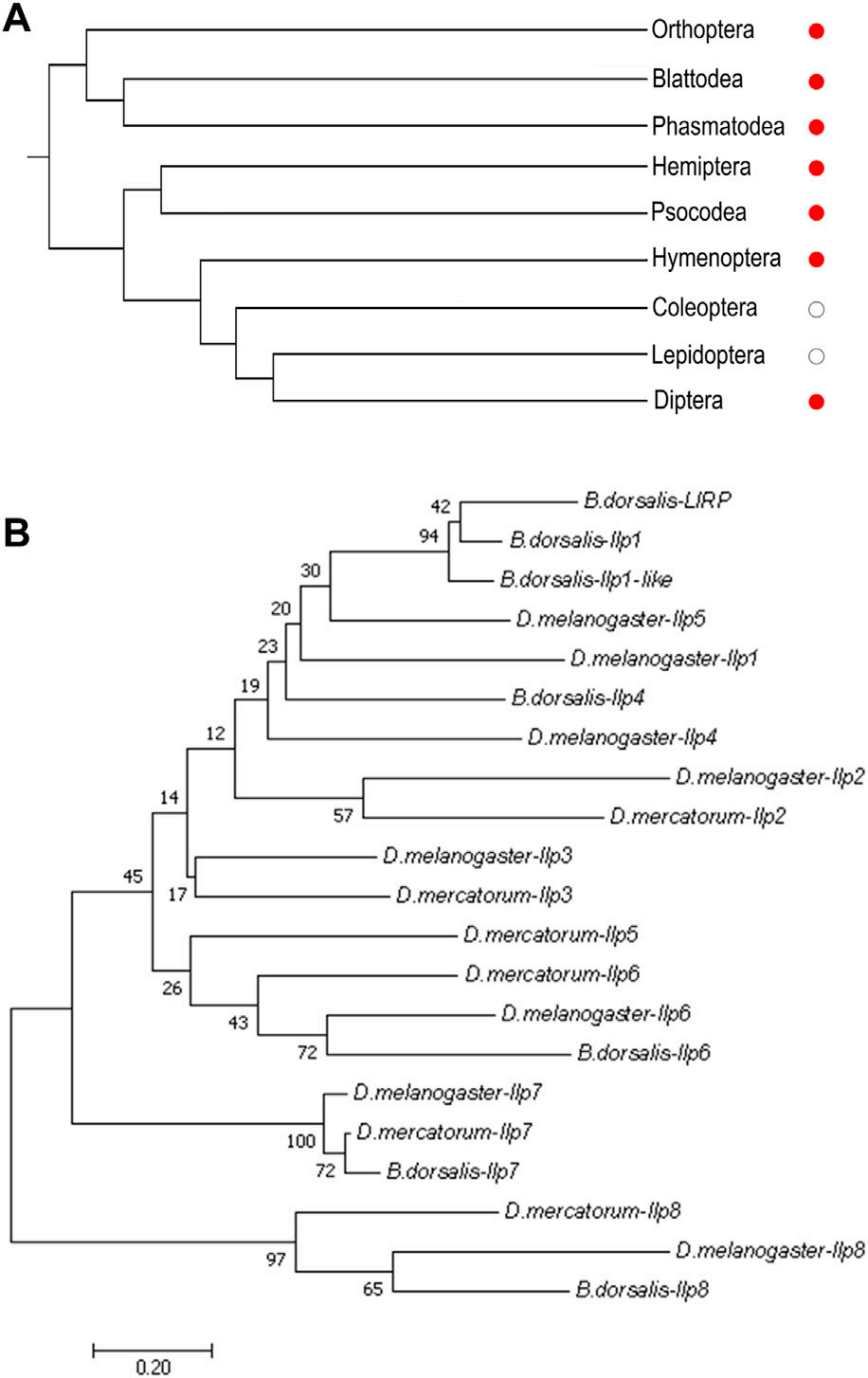
Phylogenetic analyses of insect Ilp8s. **(A)** Cladogram shows that orthologs of Ilp8s are found in major groups of insects (red dots) except for the lepidopterans and coleopterans (white dots). **(B)** Phylogenetic tree of Ilps in *D. melanogaster*, *D. mercatorum* and *B. dorsalis*. The Ilp8 cluster is well separated from the other fly Ilps. The neighbor-joining tree was generated using MEGA 7.0 with 1,000 bootstrap test. Bootstrap values are indicated at each branch. The scale bar represents the branch length.

When currently available genomic and transcriptomic data were analyzed, genes encoding orthologs of Ilp8s were found in major groups of insects except for the lepidopterans and coleopterans (Figure 1A; Supplementary Data Sheet S1). The amino acid sequences of Ilps in three dipteran insects, the fruit fly (*D. melanogaster*), the fruit fly’s distantly related species *Drosophila mercatorum*, and the oriental fruit fly (*Bactrocera dorsalis*) were isolated (Supplementary Data Sheet S1) for further analysis. There are 8 Ilps in fruit fly (dIlp1-8), while a total of 6 Ilps are identified in *D. mercatorum* (DrIlps) and 7 Ilps are found in *B. dorsalis* (BdIlps). When a neighbor-joining phylogenetic tree was constructed, the Ilp8s were found to be well separated from other Ilps in these three species (Figure 1B).

**FIGURE 2:**
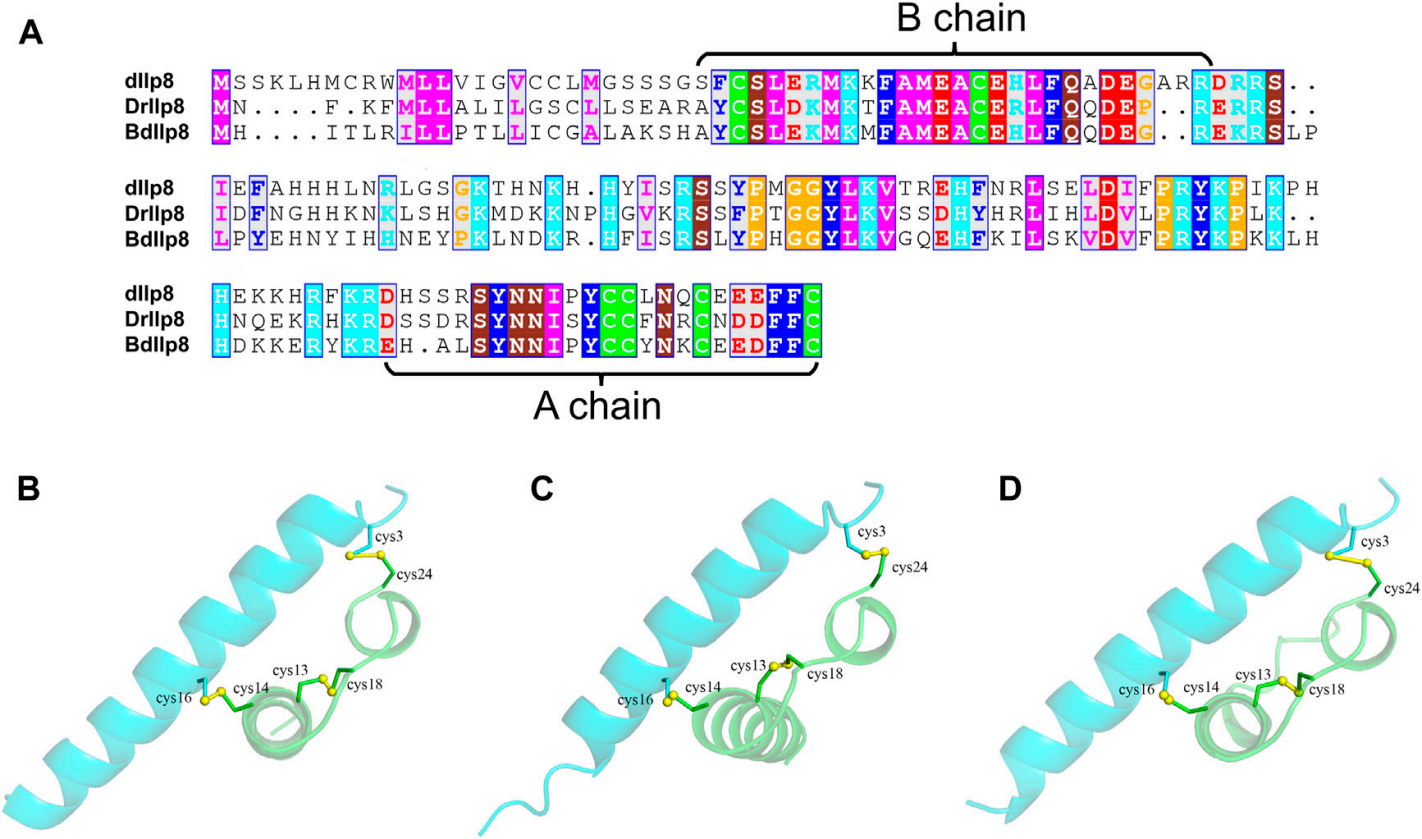
The sequence and structure homology of fly Ilp8s. **(A)** Amino acid sequence alignment of fly Ilp8s. The six conserved cysteine residues are highlighted in green, and other conserved residues are visualized by different colors. The fragments corresponding to the A chain and B chain of mature Ilp8s are labeled with curly braces. **(B–D)** The predictive three-dimensional structure models of the *D. melanogaster* **(B)**, *D. mercatorum* **(C)** and *B. dorsalis* **(D)** mature Ilp8 peptides. The structure is colored as follows: A chain in green, B chain in light blue and disulfide bonds between cystine residues in yellow.

Fairly large variations were noticed when the amino acid sequences of Ilp8 in representative insect species were compared with each other (Figure 2A), which is consistent with previous reports (Gontijo and Garelli, 2018; Veenstra, 2020). Among three fly species, sequence alignment showed that DrIlp8 and BdIlp8 shares 54.93% and 53.42% identities with dIlp8, respectively (Table 1). The insulin peptides are normally hydrolyzed into two polypeptide chains (A chain and B chain), which are then linked together by two disulfide bonds to form the mature product (Steiner et al., 2009). A motif of six cysteine residues crucial for the formation of disulfide bridges is the key signature of Ilps (Nili et al., 2012). These six cysteine residues were found to be highly conserved in the fly Ilp8 peptides (Figure 2A). When three-dimensional structure models of the fly Ilp8s were generated, a conserved tertiary structure was evident (Figures 2B–D). Fly Ilp8s were predicated to contain two alpha helices and an intrachain disulfide bond in the A chain, and a long alpha helix in the B chain. The A chain and B chain of fly Ilp8s are likely connected *via* two interchain disulfide bonds (Figures 2B–D). Amino acid sequence alignment indicates that the Lgr3 receptor is also quite conserved in these fly species (Supplementary Figure S1). The similarity of signature motif, predictive structure and receptor sequence among the fly Ilp8 peptides suggest that they might function in conserved fashions.

**TABLE 1:** Sequence similarity of fly Ilp8s.

Protein name	Accession	Protein length	Similarity (dIlp8)
dIlp8	NP_648949.2	150aa	100%
DrIlp8	From trinity	142aa	54.93%
BdIlp8	XP_011201638.2	145aa	53.42%

### Fly Ilp8s are primarily expressed in the ovary

**FIGURE 3:**
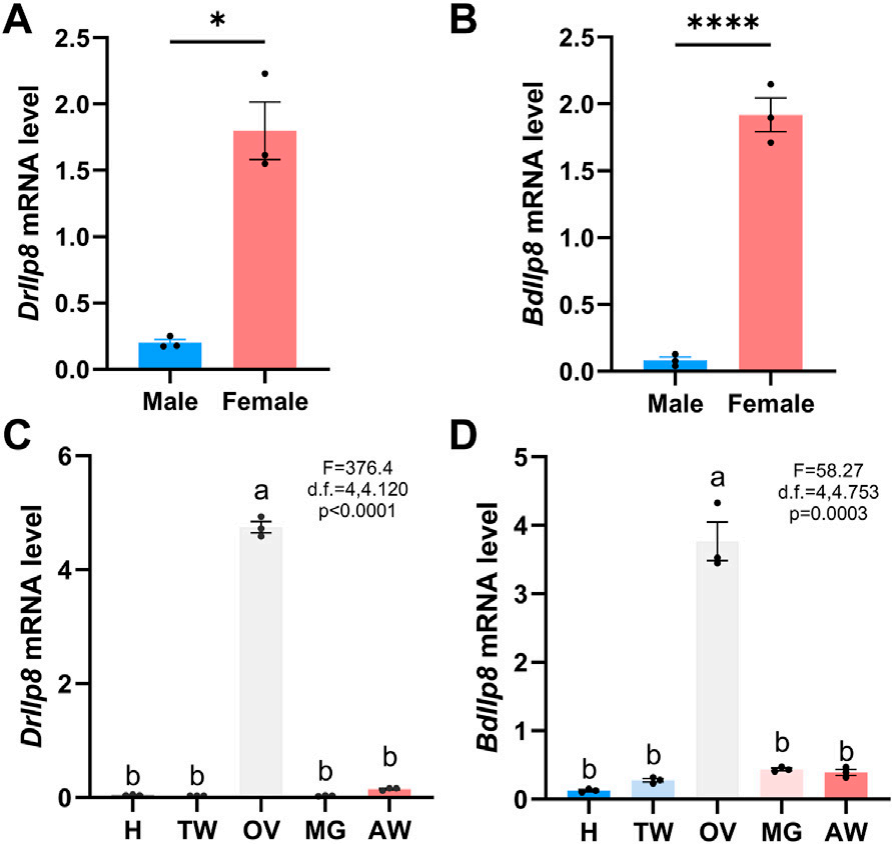
Fly Ilp8s are primarily expressed in the ovary. **(A)** The mRNA level of *DrIlp8* was significantly higher in the females (Welch’s *t*-test, *p* < .05). **(B)** The mRNA level of *BdIlp8* was significantly higher in the females (Welch’s *t*-test, *p* < .001). **(C)** Distribution of *DrIlp8* transcripts in different tissues. Head (H), thorax without midgut (TW), ovary (OV), midgut (MG) and abdomen without midgut and ovary (AW) were included. The mRNA level of *DrIlp8* was significantly higher in the ovary (*p* < .0001). **(D)** The mRNA level of *BdIlp8* was significantly higher in the ovary (*p* = .0003). Different letters indicate significant differences between groups using Welch’s ANOVA test with Turkey multiple comparisons **(C,D)**.

Most Ilps are produced by neuroendocrine cells, but Ilp8s are primarily expressed in gonads in various insect species (Veenstra, 2020). When examined by real-time quantitative PCR, both *DrIlp8* and *BdIlp8* were found to be expressed in the adult females at significantly higher level than that in males (Figures 3A, B). Based on the sexual dimorphic expression profile, the distribution of *Ilp8* in different tissues were further examined in female flies. In *D. mercatorum*, *Ilp8* was expressed at a very high level in the ovary, with very low levels in the head, thorax, midgut and abdomen (Figure 3C). Similarly, the expression level of *BdIlp8* in the ovary was significantly higher than that in other tissues in *B. dorsalis* (Figure 3D). The enriched expression in ovary indicates that *Ilp8* might be involved in female reproduction, which was further tested by dsRNA knock-down experiments.

### Fly Ilp8s are required for female fecundity

**FIGURE 4:**
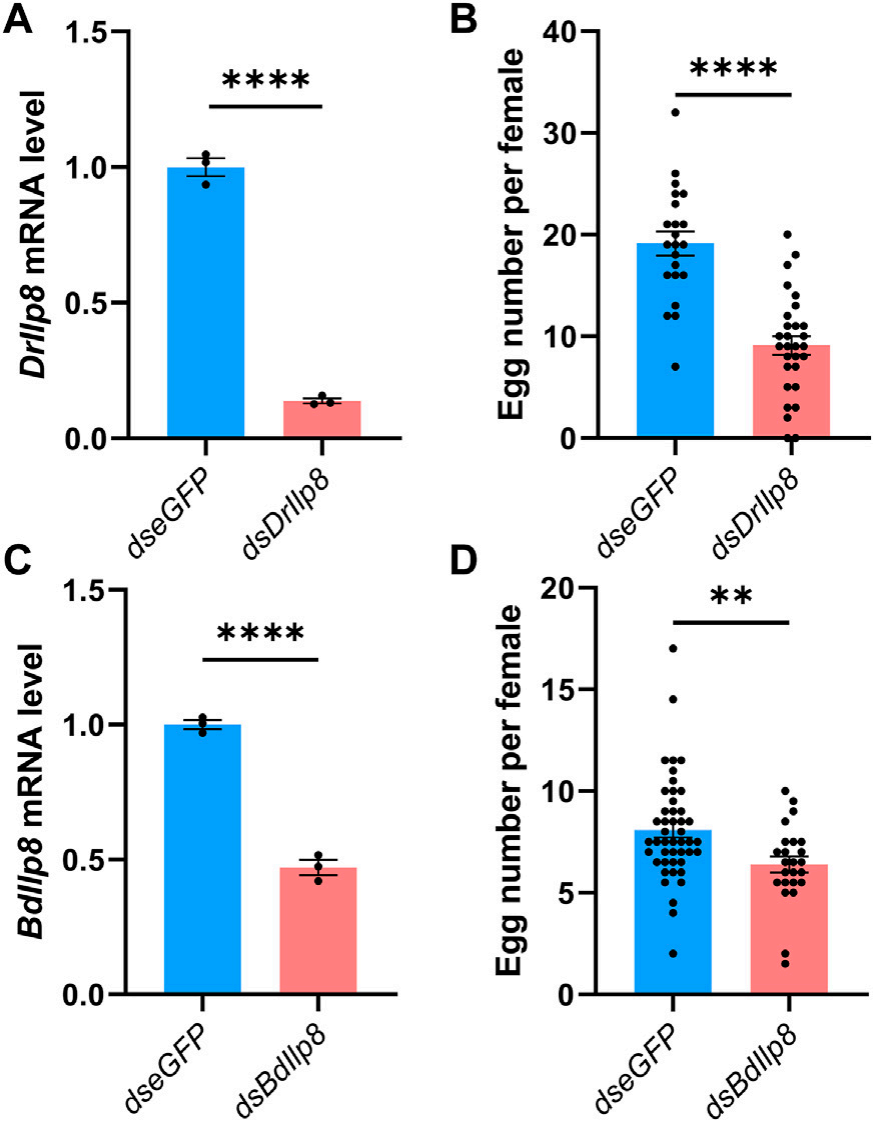
Fly Ilp8s are required for female fecundity. **(A)** The *DrIlp8* mRNA level was downregulated after ds*DrIlp8* injection (Welch’s *t*-test, *p* < .001). **(B)** The number of eggs laid by single young mated female flies in a period of 24 h was significantly reduced after *dsDrIlp8* treatment (Welch’s *t*-test, *p* < .001). **(C)** The *BdIlp8* mRNA level was downregulated after *dsBdIlp8* injection (Welch’s *t*-test, *p* < .001). **(D)** The number of eggs laid by single young mated female flies in a period of 24 h was significantly reduced after *dsBdIlp8* treatment (Welch’s *t*-test, *p* < .01). Values and error bars represent the mean and SEM of three independent biological replicates.

Specific dsRNAs targeting *DrIlp8* and *BdIlp8* (Supplementary Figure S2A; Supplementary Data Sheet S2) were synthesized and injected into female flies, and dsRNA against *eGFP* was used as control. The mRNA level of *DrIlp8* was strongly downregulated to 14% of the control group after dsRNA injection (Figure 4A). When the number of eggs laid by female flies was calculated, a drastic reduction of fecundity was observed (Figure 4B). Similarly, inhibiting *BdIlp8* expression by dsRNA (Figure 4C) also significantly impaired the fecundity (Figure 4D). Thus, Ilp8 are required for female fecundity in both *D. mercatorum* and *B. dorsalis.*

### Fruit fly Ilp8 regulates female sexual attractiveness

**FIGURE 5:**
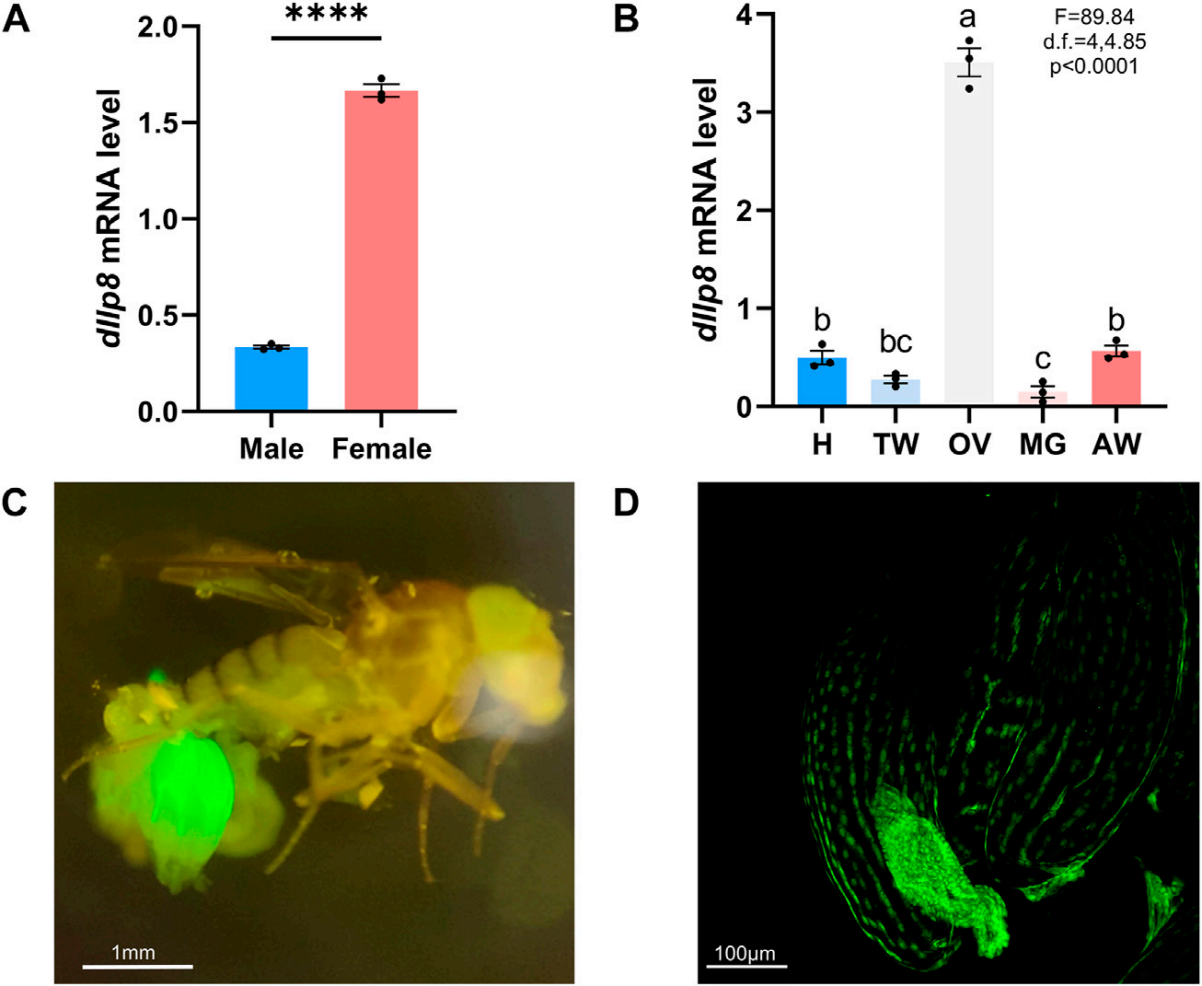
The expression pattern of fruit fly *Ilp8*. **(A)** The mRNA level of *dIlp8* was significantly higher in the females (Welch’s *t*-test, *p* < .001). **(B)** The expression of *dIlp8* was highly elevated in the ovary (Welch’s ANOVA test, *p* < .0001). Different letters indicate significant differences between groups using Welch’s ANOVA test with Turkey multiple comparisons. **(C)** The dIlp8-GFP reporter was mainly activated in the ovary. **(D)** The dIlp8-GFP reporter were expressed in the follicle cells surrounding late stage oocytes and in the corpus luteum.

The model insect fruit fly was further utilized to investigate the role and mode of action of Ilp8 during reproduction. When examined by qRT-PCR, *dIlp8* was found to be expressed at a significantly higher level in adult females than in males (Figure 5A). Examination of tissue expression profiles showed that *dIlp8* was expressed at a very high level in the ovary, with very low levels in the head, thorax, midgut and abdomen (Figure 5B). The expression pattern of *dIlp8* was visualized using a GFP reporter line (Supplementary Figure S2B), in which the expression of GFP is under control of the endogenous genomic regulatory elements of *dIlp8* (Garelli et al., 2012). Strong green fluorescence was found in the abdomen of female flies, which was mainly restricted in the ovaries (Figure 5C). At a closer observation, GFP was found in the follicle cells surrounding late stage oocytes, as well as in the corpus luteum which consists of follicle cells trimmed and left over after ovulation (Figure 5D). The expression of d*Ilp8* in female flies was found to be downregulated after successful mating (Supplementary Figure S2C). The reproduction organ specific and mating status responsive expression pattern of *dIlp8* is suggestive for its involvement in female reproduction.

**FIGURE 6:**
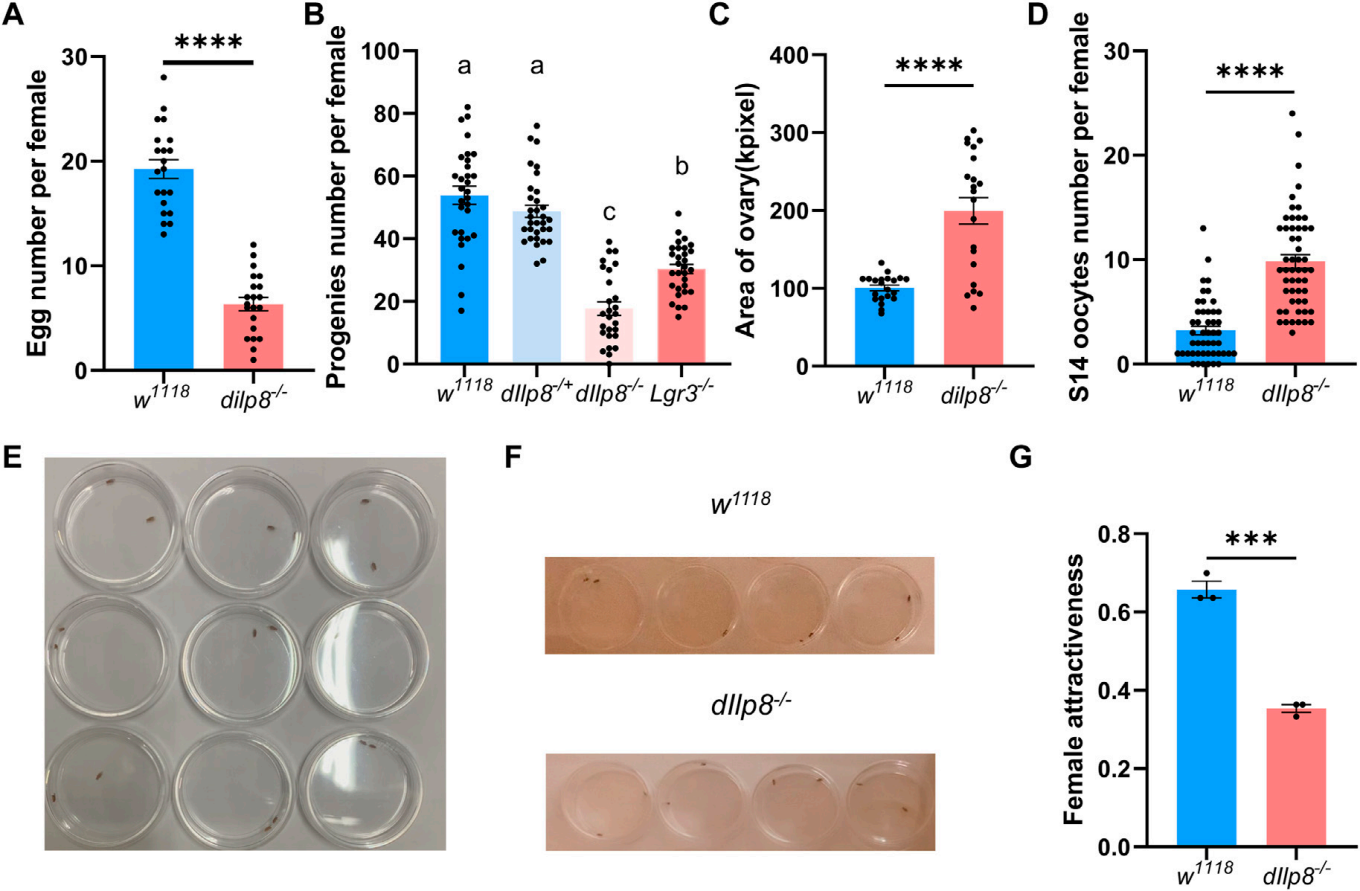
dIlp8 regulates female fecundity and sexual attractiveness. **(A)** *dIlp8* ^*−/−*^ females laid significantly fewer eggs than wild type flies (Welch’s *t*-test, *p* < .01). **(B)** *dIlp8* ^*−/−*^ and *Lgr3* ^*−/−*^ females produced significantly decreased number of progenies than wild type flies (Welch’s ANOVA test). **(C)** *dIlp8* ^*−/−*^ females showed much larger ovary than wild type files (Welch’s *t*-test, *p* < .001). **(D)** *dIlp8* ^*−/−*^ females retained significantly increased amount of stage 14 mature eggs (Welch’s *t*-test, *p* < .001). **(E)** Set up of the courtship chambers. **(F)** The *dIlp8* ^*−/−*^ females were less attractive than wild type flies as shown by screenshots from supplemental videos. **(G)** Sexual attractiveness of *dIlp8* ^*−/−*^ females were significantly reduced (Welch’s *t*-test, *p* < .005). Three biological replicates were included, and each replicate group contains ten pairs of flies.

The role of *dIlp8* in female fecundity was tested using the loss-of-function mutant strain *dIlp8* ^*MI00727*^ (Garelli et al., 2012). The *dIlp8* ^*MI00727*^ homozygous (*dIlp8* ^*−/−*^) females laid significantly fewer number of eggs than wild type flies (Figure 6A). However, no obvious developmental defects were observed in the ovary of *dIlp8* ^*−/−*^ virgin females (Supplementary Figures S2D, E), indicating that dIlp8 likely impacts female fecundity without modulating reproduction system development. Mutation of both *dIlp8* and its receptor *Lgr3* resulted in reduction of the number of progenies produced by female flies (Figure 6B), implicating that the dIlp8-Lgr3 signaling cascade operates in female fecundity regulation. We noticed that the abdomen of *dIlp8* ^*−/−*^ females are often swollen when rearing with wild type males for 2 weeks, which is likely due to enlargement of the ovaries (Figure 6C). In *dIlp8* ^*−/−*^ flies, the number of stage-14 mature oocytes that were retained in the ovary was higher than that of the wild type flies (Figure 6D). These observations suggest that *dIlp8* ^*−/−*^ flies are defective in ovulation, which may contribute to the decrease of fecundity.

Our finding that dIlp8 might modulate ovulation is consistent with previous study (Liao and Nässel, 2020), but the underlying mechanism is not fully understood. It has been shown that successful mating with males strongly stimulates ovulation in female flies (Rubinstein and Wolfner, 2013). The potential role of dIlp8 in mating success was further examined by behavior analysis. Pairs of single virgin female and healthy male were individually placed in courtship chambers and the courtship processes were videotaped for analysis (Figure 6E). Unexpectedly, the sexual attractiveness of *dIlp8* ^*−/−*^ female flies were severely weakened, evidenced by the reduced tendency of males to initiate courtship (Figures 6F, G). These observations indicate that *dIlp8* ^*−/−*^ female flies are sexually less attractive, which result in decreased mating success followed by ovulation defects and fecundity reduction. After knocking-down *DrIlp8* by dsRNA in *D. mercatorum*, the attractiveness of female flies was reduced but the difference was not statistically significant (Supplementary Figures S3A, B). It is possible that DrIlp8 is not crucial for female attractiveness in *D. mercatorum,* but we believe that the efficiency of RNAi needs to be further increased to settle the dispute. Ultimately, generation and examination of *DrIlp8* and *BdIlp8* loss-of-function mutants will provide direct evidence regarding the role of Ilp8 in female fecundity and sexual attractiveness.

### Transcriptomic analysis of *dIlp8* mutant females

**FIGURE 7:**
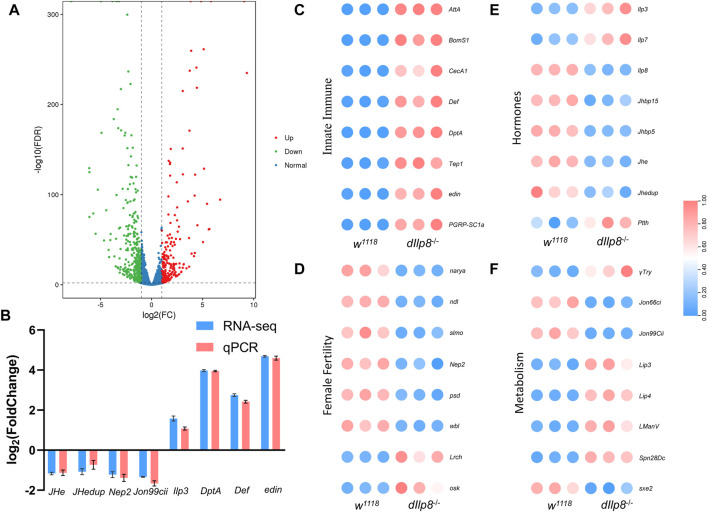
RNA-seq analysis of *dIlp8* mutant fly. **(A)** Volcano plot of differently expressed genes in *dIlp8* ^*−/−*^ virgin females. A total of 667 genes were differentially expressed, among which 410 genes were downregulated (green dots) and 257 genes were upregulated (red dots). **(B)** The mRNA levels of representative DEGs were examined by qRT-PCR (red columns) and compared with the RNA-seq data (blue columns). **(C)** Heatmap of the innate immune signaling pathway. **(D)** Heatmap of genes with known roles in female fertility. **(E)** Heatmap of genes involved in steroid hormones biosynthesis. **(F)** Heatmap of metabolism genes. The expression level of all genes was normalized by zero-to-one method.

To get a more comprehensive view of the effects upon loss of *dIlp8*, transcriptomic analysis was performed to compare the changes of gene expression between *dIlp8* ^*−/−*^ and wild type virgin females. A total of 667 genes were differentially expressed in *dIlp8* ^*−/−*^ flies (Supplementary Data Sheet S3), among which 410 genes were downregulated and 257 genes were upregulated (Figure 7A). The mRNA level of various representative genes (*JHe*, *JHedup*, *Nep2*, *Jon99cii*, *Ilp3*, *DptA, Def* and *edin*) were examined by qRT-PCR, and the change of the abundances of these transcripts were consistent with the RNA-seq results (Figure 7B). Further analysis found that loss of *dIlp8* strongly influenced the following biological processes: lysosome-related catabolism, defense mechanisms, insect hormone metabolism and oocyte development (Figures 7C–F; Supplementary Figures S3C). Thus, it appears that various developmental, physiological and metabolic factors related with female fecundity are mis-expressed in *dIlp8* ^*−/−*^ flies. In *D. mercatorum* and *B. dorsalis*, several genes including *DrJon99cii*, *BdNep2* and *BdWbl* were affected in a similar fashion after knocking-down *Ilp8* by dsRNA (Supplementary Figures S3D–F). These observations imply that Ilp8 might modulate female fecundity through similar pathways in these fly species.

## Discussion

Our present results suggest that Ilp8s are functionally conserved among three fly species. Despite the significant differences of the amino acid sequences, the predicted structure, the expression pattern and the role in female fecundity of Ilp8 in three fly species are very consistent. We provided further evidence to demonstrate a novel role of dIlp8 in female sexual attractiveness which likely underlies the observed ovulation and fecundity defects in the *dIlp8* mutants. Given that dIlp8 are specifically expressed in the follicle cells of late stage oocytes, it is tempting to speculate that dIlp8 might serve as an interorgan communication signal to coordinate the sexual behavior with oocyte development. Mutation of *dIlp8* leads to mis-regulation of genes involved in various biological aspects, including the steroid hormones biosynthesis and metabolism pathways. It has been shown that the dIlp8-Lgr3 signaling circuit triggers hormonal responses and impacts the activity of several crucial insect hormones such as insulin, prothoracicotropic hormone and ecdysone in the larval stage (Garelli et al., 2012; Garelli et al., 2015; Vallejo et al., 2015). In the adult stage, the steroid hormones (Soller et al., 1999; Bilen et al., 2013; Chiang et al., 2016) as well as the insulin pathway (Fedina et al., 2012; Kuo et al., 2012; Lebreton et al., 2015; Fedina et al., 2017; Lin et al., 2018) have important influence on female sexual attractiveness. Whether dIlp8 affects the activity of these pathways in a similar fashion and whether dIlp8 regulates female attractiveness through these pathways will be further investigated.

In female fruit flies, ovary development and maturation are tightly controlled by varying environmental and physiological conditions, which are sensed and processed by organs such as brain, gut and fat body (Drummond-Barbosa, 2019). These tissues employ metabolites, hormones, and other factors to regulate ovary development, but how ovary provides feedback to other organs is still poorly understood (Drummond-Barbosa, 2019; Okamoto and Watanabe, 2022). It has been known for a long time that the steroid hormone ecdysone is primarily produced by the ovary in adult female flies (Bownes and Smith, 1984), but the target organs and modes of action of ovary generated ecdysone have only began to be identified recently (Sieber and Spradling, 2015; Ameku and Niwa, 2016; Baron et al., 2018; Ahmed et al., 2020; Zipper et al., 2020). Whether ovary can utilize Ilp8 as another signaling molecule to ensure successful reproduction needs further explorations.

The dIlp8-Lgr3 pathway plays crucial role in coordinating imaginal tissue growth with developmental transitions (Gontijo and Garelli, 2018). In response to growth perturbations, the Ilp8-Lgr3 pathway adjusts developmental timing to allow regeneration of damaged tissues and to coordinate growth of body parts (Colombani et al., 2012; Garelli et al., 2012; Garelli et al., 2015; Vallejo et al., 2015). Such error sensing and repairing mechanisms ensures developmental stability when facing accidental as well as stochastic perturbations. It has been known for decades that similar regeneration promoting and growth coordinating mechanism exists among cockroaches, moths and flies, which represent distinct insect development modes (Stock’and O'Farrell, 1954; Madhavan and Schneiderman, 1969; Dewes, 1973; Kunkel, 1977; Simpson et al., 1980; Poodry and Woods, 1990; Smith-Bolton et al., 2009; Jaszczak and Halme, 2016). Whether the Ilp8-Lgr3 pathway is commonly used in different insect species to safeguard development stability remains an open question. Our finding that *Lgr3* mutant shows similar female fecundity defect indicates that the same signal transduction cascade might be utilized in the adulthood for a novel purpose. Interestingly, it has been shown that activation of a subset of neurons expressing Lgr3 in the adult females reduces their sexual receptivity to male courtship and also leads to decreased fecundity (Meissner et al., 2016). It is possible that the Ilp8-Lgr3 signaling plays a dosage sensitive role in female fecundity.

It is interesting to notice that orthologs of Ilp8 could be found in major groups of insects except for the moths, butterflies and bettles (Figure 1A; Supplementary Table S1). We suspect that one or more members of the remarkably expanded Ilp family in moths and bettles may have evolved to replace the functions of Ilp8. However, it seems difficult to interpret the absence of Ilp8 and Lgr3 in mosquitoes (Gontijo and Garelli, 2018). The symbol of Ilp8 has been assigned to an Ilp gene in *Aedes aegypti* (Ling et al., 2017)*,* but sequence similarity analysis indicates it is likely an ortholog of dIlp1. In *A. aegypti*, both Ilp7 and Ilp8 regulate ovarian development, but they are specifically expressed in the head without detectable expression in the ovary (Ling et al., 2017). The ortholog of Ilp8 receptor Lgr3 is also absent in *A. aegypti* (Brown et al., 2008). Clearly, more work is required to understand the multifaceted functions and the evolution of the Ilp8-Lgr3 pathway in insects.

## Materials and methods

### Insect rearing

The *D. melanogaster* stocks *w* ^*1118*^ (#3605), Canton S (#64349) and *dIlp8* ^*MI00727*^ (#33079) were obtained from the Bloomington Drosophila Stock Center. The *Lgr3* ^*KO*^ mutant (*Lgr3* ^*−/−*^, Deng et al., 2019) is a gift from Dr. Yi Rao. The *w* ^*1118*^ strain was used to examine *dIlp8* expression pattern (Figures 5A,B) and used as control in female fecundity (Figure 6) as well as transcriptomic analysis (Figure 7). The Canton S males were used in female fecundity assays (Figure 6). The *D. mercatorum* strain (#1525.49) was obtained from the National Drosophila Species Stock Center at Cornell University. Both *D. melanogaster* and *D. mercatorum* stocks were maintained on standard fly food in 25°C incubators as described previously (Wang et al., 2019; Mo et al., 2020). The *B. dorsalis* stock flies were originally collected from a citrus orchard in Yunnan Province of China in 2020 and have been reared for several generations in the laboratory. Adult flies of *B. dorsalis* were held in 40 cm × 30 cm × 30 cm cages and had free access to a solid artificial diet (Xu et al., 2015). The *B. dorsalis* insects were maintained at 25 ± 1°C, 65 ± 5% relative humidity and a 14:10 h (light: dark) photoperiod.

### Bioinformatic analyses

The sequences of fly Ilp peptides (Supplementary Data Sheet S1) were aligned with MAFFT version 7 (Katoh et al., 2019) and edited manually by BioEdit. A molecular phylogenetic tree was constructed by the neighbor joining method using a Poisson model with a bootstrap of 1,000 replicates in MEGA7 (Kumar et al., 2016). The three-dimensional structure models of the fly Ilp8 peptides were constructed by Alpha Fold Colab (https://colab.research.google.com/github/sokrypton/ColabFold/blob/main/AlphaFold2.ipynb, number of recycles = 3).

### Quantitative real-time PCR

Whole flies were flash frozen in liquid nitrogen before homogenate were prepared. Three days old virgin females and males of *D. mercatorum* were used to examine the expression of *DrIlp8* in whole flies (Figure 3A), and 10 days old *B. dorsalis* virgin females and males were used to examine *BdIlp8* expression pattern (Figure 3B). Three days old virgin females and males of *w* ^*1118*^ fruit flies (Figure 5A) were used to examine *dIlp8* expression in the whole body. For the tissue-specific gene expression analysis, tissues of fly head (H), thorax without midgut (TW), ovary (OV), midgut (MG) and abdomen without midgut and ovary (AW) were dissected in cold PBS and collected. Three days old virgins of *D. mercatorum* (Figure 3C) and *w* ^*1118*^ fruit flies (Figure 5B) were collected for tissue sampling. Ten days old *B. dorsalis* virgin females were used to examine the tissue specific expression pattern of *BdIlp8* (Figure 3D).

Total RNAs were extracted using the TianGen RNA isolation kit (TianGen, Beijing, China). The quantity and quality of RNA were assessed by agarose gel electrophoresis and the Nanodrop 2000 micro-spectrophotometer (Thermo, United States). Equal amounts (1 μg) of RNA were used as the template in the reverse transcription reaction using the HiScript III All-in-one RT SuperMix Perfect for qPCR (Vazyme, Nanjing, China).

Quantitative real-time PCR (qRT-PCR) experiments were carried out in an ABI QuantStudio 6 Flex platform (Thermo, United States) using the Taq Pro Universal SYBR qPCR Master Mix (Vazyme, Nanjing, China). Reactions were performed in a 10 μl mixture containing 5 μl Master Mix, 2 pmol of each primer, 1 μl cDNA sample and nuclease-free water. The sequences of primers used for qRT-PCR were listed in Supplementary Data Sheet S2. The mRNA levels were normalized to the internal reference gene *GAPDH* (for *D. mercatorum*) and Rpl32 (for *D. melanogaster* and *B. dorsalis*) using the 2^−ΔΔCT^ method (Livak and Schmittgen, 2001; Cao et al., 2021).

### RNA interference

The DNA fragments covering the target region were amplified from Ilp8 mRNAs by RT-PCR, and T7 promoter sequences were added onto the primers (Supplementary Data Sheet S2). The DNA fragments were purified and sequenced before used as the templates to generate dsRNAs. Double strand RNA (dsRNA) against *D. mercatorum* and *B. dorsalis Ilp8* were generated using the T7 RiboMAX™ Express RNAi System (Promega, United States). The length of dsRNA is 395bp for *DrIlp8* and 341bp for *BdIlp8* (Supplementary Figure S2A). The dsRNAs were purified and examined by gel electrophoresis, and the concentrations were quantified using Nanodrop. The dsRNA against *eGFP* (enhanced Green Fluorescent Protein) was used as control. The dsRNAs were diluted to 1 μg/μl for microinjection. Three days old virgin females of *D. mercatorum* were anesthetized by carbon dioxide, and 200 ng dsRNA was injected into the ventral abdomen of each fly. Ten days old virgin females of *B. dorsalis* were immobilized on ice, and 1 μg of dsRNA was injected into the ventral abdomen of each insect. A total of fifty flies were used for each dsRNA treatment. The expression level of *Ilp8* was examined by qRT-PCR at 24 h after dsRNA injection in *D. mercatorum*, and at 48 h after dsRNA injection in *B. dorsalis*.

### Imaging

Female fruit flies were dissected in cold PBS, and ovaries were washed two times with PBS. Figures were captured under stereo microscope equipped with fluorescence adapter SFA-LFS-GR (Nightsea, United States) and an inverted fluorescence microscope (AMG EVOS, ThermoFisher, United States).

### Fecundity and sexual behavior measurements

Fecundity was measured by calculating the number of eggs produced by young mated female flies over a 24-h laying period. One virgin female was kept with two healthy males for 3 days before transferred to a new vial without anesthesia. The flies were removed from the vials and the number of eggs was recorded per 24 h (Figures 4B, 4D, 6A). For *D. melanogaster* studies, the number of progenies produced by single young female flies in a period of 3 days after mating with two healthy males were counted (Figure 6B).

To examine the sexual behavior of fruit flies, one virgin female was crossed with one male in a courtship chamber. The mating processes were recorded for 2 h and the mating behaviors were manually analyzed. Three biological replicates were included, and each replicate group contains ten pairs of flies.

### Statistics

Graphpad prism 8.0.2 (GraphPad Software, United States, www.graphpad.com) was used for statistical analyses. Values and error bars represent the mean and SEM of independent biological repeats. Experimental data with just two groups were analyzed using the Student’s or Welch *t*-test. Experimental data with more than three groups were analyzed by Welch one-way ANOVA test with Tukey HSD multiple comparison.

### RNA sequencing

Total RNAs were extracted from three replicates group of 3 days old virgin females of *w* ^*1118*^ and *dIlp8* ^*−/−*^ stocks. The quantity and quality of RNA have been assessed by Nanodrop, Qubit 2.0 and Agilent 2100. Magnetic beads with oligo (dT) were performed to enrich Poly (A) mRNA from total RNA. The cDNA library was constructed using the Paired-End Sample Preparation Kit (Illumina Inc., San Diego, CA, United States) followed the manufacturer’s instructions. To ensure quality control, Qubit 2.0, Agilent 2100 and quantitative Real-Time PCR were performed, and then the transcriptome sequencing was carried out using IlluminaHiSeq 6000 with PE100 approach (Biomarker Technology Corporation, Beijing, China). The clean reads were filtered from raw data by removing adaptor sequence, primer reads and low-quality bases. We use the longest isoform to represent a gene for downstream analysis. The reads of each sample were mapped to the reference genome of *D. melanogaster.* The gene abundance was represented by PKM value. DEG screening in sample group was conducted with the DEGseq package. False discovery rate (FDR) value ≤ .01 and fold change (FC) ≥ 2 in the Benjamini and Hochberg method were chosen as DEGs.

## Data Availability

The datasets presented in this study can be found in online repositories. The names of the repository/repositories and accession number(s) can be found below: NCBI Sequence Read Archive (SRA) SRR22543184, SRR22543185, SRR22543186, SRR22543187, SRR22543188 and SRR22543189.
