# Supplementary figures and images for: Insulin-like peptide 8 (Ilp8) regulates female fecundity in flies
Source: Front Cell Dev Biol. 2023 Jan 18;11:1103923. doi: 10.3389/fcell.2023.1103923 (PMC9890075; doi:10.3389/fcell.2023.1103923)

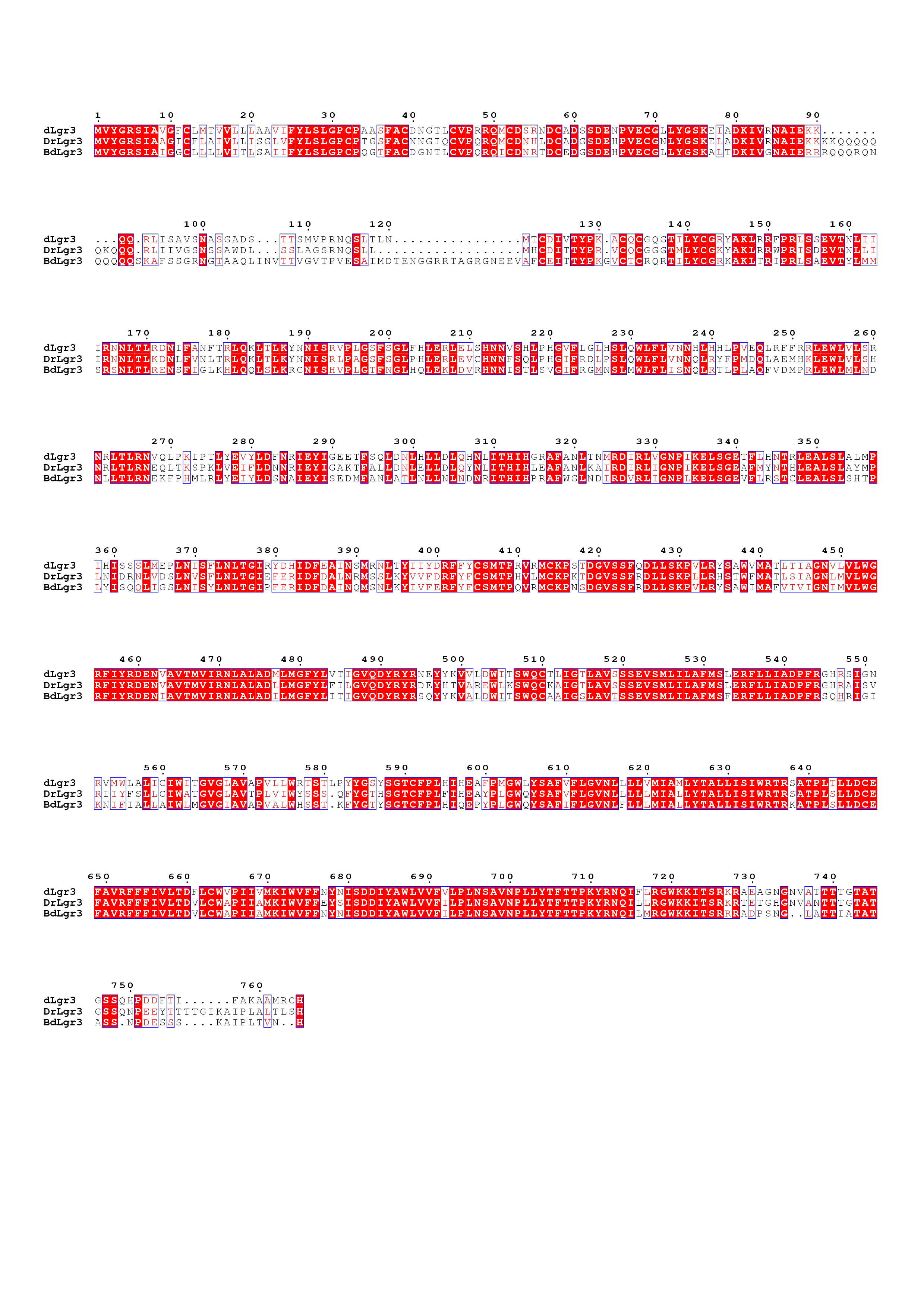

Supplement: Supplementary file 2 [file Image1.TIFF]
