# Supplementary material for: Insulin-like peptide 8 (Ilp8) regulates female fecundity in flies
Source: Front Cell Dev Biol. 2023 Jan 18;11:1103923. doi: 10.3389/fcell.2023.1103923 (PMC9890075; doi:10.3389/fcell.2023.1103923)
Supplement: Supplementary file 7 [file Table4.DOCX]

**Figure Legends of Supplementary Figures**

**Figure S1.** Amino acid sequence alignment of fly Lgr3 receptors. The completely conserved residues are shadowed in red, and other conserved residues are visualized by different colors.

**Figure S2.** **(A)** Target region of dsDrIlp8 and dsBdIlp8. The length of mRNAs including UTR regions is shown, and the target regions are marked with red brackets. **(B)** The schematic diagram for the dIlp8-GFP reporter line. The eGFP cassette is inserted in the first intron of the dIlp8 genomic region. **(C)** The expression of *dIlp8* is significantly down-regulated after mating in *D. melanogaster* (Welch’s t-test, p<0.05). **(D-E)** The morphology of female reproduction system in wild type **(D)** and *dIlp8^-/-^* mutant **(E)** flies. Three days old virgins were collected and the ovaries were dissected.

**Figure S3.** **(A)** The *DrIlp8* mRNA level was down-regulated after dsDrIlp8 injection (Welch’s t-test, p<0.05). **(B)** The attractiveness of *D. mercatorum* females was not significantly reduced after dsDrIlp8 injection. **(C)** Loss of *dIlp8* resulted in reduction of the expression of several genes regulating oocyte development. **(D)** The mRNA level of *DrJon99cii* was down-regulated after dsDrIlp8 injection (Welch’s t-test, p<0.05). **(E)** The mRNA level of *BdIlp8* was down-regulated after dsBdIlp8 injection (Welch’s t-test, p<0.05). **(F)** The mRNA level of *BdWbl* and *BdNep2* were down-regulated after dsBdIlp8 injection (Welch’s t-test, p<0.01 and p<0.05).
